# Calcium–Permeable Channels and Endothelial Dysfunction in Acute Lung Injury

**DOI:** 10.3390/cimb44050150

**Published:** 2022-05-16

**Authors:** Ying Hao, Zhuang Wang, Francis Frimpong, Xingjuan Chen

**Affiliations:** 1 College of Sports, Northwest Normal University, Lanzhou 730070, China; haoying@nwnu.edu.cn (Y.H.); rimpfrank@gmail.com (F.F.); 2 Institute of Medical Research, Northwestern Polytechnical University, Xi’an 710072, China; todd1997@mail.nwpu.edu.cn

**Keywords:** calcium channels, endothelial cells, lung injury, permeability

## Abstract

The increased permeability of the lung microvascular endothelium is one critical initiation of acute lung injury (ALI). The disruption of vascular-endothelium integrity results in leakiness of the endothelial barrier and accumulation of protein-rich fluid in the alveoli. During ALI, increased endothelial-cell (EC) permeability is always companied by high frequency and amplitude of cytosolic Ca^2+^ oscillations. Mechanistically, cytosolic calcium oscillations include calcium release from internal stores and calcium entry via channels located in the cell membrane. Recently, numerous publications have shown substantial evidence that calcium-permeable channels play an important role in maintaining the integrity of the endothelium barrier function of the vessel wall in ALI. These novel endothelial signaling pathways are future targets for the treatment of lung injury. This short review focuses on the up-to-date research and provide insight into the contribution of calcium influx via ion channels to the disruption of lung microvascular endothelial-barrier function during ALI.

## 1. Introduction

Acute lung injury (ALI) is a significant source of morbidity and mortality in various critically ill patient population, including pneumonia, non-pulmonary sepsis, inhalation of toxicants, severe trauma, shock, or other factors [1]. Pulmonary edema and hypoxic respiratory failure are the main symptoms of ALI. There are now more than 480 million COVID-19 cases worldwide (WHO Coronavirus (COVID-19) Dashboard), 10–20% of which have displayed acute lung injury (ALI) and even developed acute respiratory distress syndrome (ARDS), with an associated mortality of about 3% [2,3,4]. It is important to understand the pathogenesis of ALI for aborting this process and enhancing the repair mechanism of the lung. Acute inflammation cause disruption of the lung microvascular endothelium and alveolar epithelium, resulting in initiation of ALI. The increased permeability of lung microvascular endothelium allows the influx of the protein-rich fluid into the interstitial space and the alveoli. This event causes pulmonary edema, reduces the efficiency of lung ventilation, and even lead to respiratory failure [1].

Cellular homeostatic balance of Ca^2+^ play a key role in maintaining the endothelial-barrier integrity [5,6]. Increase in the global intracellular Ca^2+^ concentration and localized Ca^2+^ signals that occur within specialized subcellular microdomains are fundamentally important components of many signaling pathways in the endothelium [7]. The increase in Ca^2+^ is modulated by ion channel on plasma membrane and endoplasmic reticular membrane. Calcium channels on cell plasma membrane mediate the influx of Ca^2+^ from the extracellular medium. These calcium-permeable channels are activated by specific stimuli, such as voltage-gated calcium channels (VGCCs), which are gated by membrane potential, receptor-operated channels (ROC) by external specific agonists, or intracellular messengers and store-operated calcium channels (SOCs) by the exhaustion of internal Ca^2+^ stores. Intracellular Ca^2+^ homeostasis depends on the normal function of these calcium channels. Therefore, Ca^2+^ channels might be potential targets for the prevention or treatment of ALI. For instance, numerous reports have demonstrated that endothelial transient receptor potential subfamily vanilloid member 4 (TRPV4) plays a key role in vascular permeability and lung inflammation during ALI [8,9,10]. This article summarizes the latest advances in understanding the role of calcium-permeable ion channels in the modulation of lung ECs and the effect of ion-channel (dys)function on the development of ALI.

## 2. The Endothelium during Lung Injury

Acute lung injury is a syndrome of acute inflammation that initiates the disruption of lung endothelial and epithelial barriers [11]. The lung microvascular ECs play key roles in controlling the barrier integrity and function [12]. The structures of tight junctions (TJs) or adherence junctions (AJs) maintain the paracellular permeability of the endothelial barrier. The monolayer of the connecting adjacent endothelial cells restrict the transport of plasma proteins of the size of albumin from the vessel lumen to the stroma (review [13]). VE-cadherin internalization and activation of acto-myosin contractility are considered to regulate the endothelial permeability [14]. However, the pathologic mechanisms and how the ECs are implicated in ALI remain as obstacles to new and effective therapies that reduce vascular leakage. The compromised EC barrier in ALI is induced by intense inflammation and pro-inflammatory cytokines (e.g., tumor necrosis factor α (TNF-α), interleukin (IL)-1β, IL-6) and other edemagenic factors such as thrombin or platelet-activating factor. The stimulation of the endothelium triggers a cascade of vascular activation, including an increased expression of vascular adhesion molecules and an increase in endothelial permeability [15,16]. Thus, the endothelium is considered as both a sensor of stimulation and a mediator of organ injury during ALI.

Toll-like receptor 4 (TLR4) in the vascular endothelium can be activated by lipopolysaccharides (LPSs), one of the bacterial endotoxins. Mice that exclusively express TLR4 on ECs (_EC_TLR4 mice) can also detect and clear intraperitoneal E. coli infection as rapidly as wild-type mice [17], indicating that the endothelial TLR4 can sense and clear intravascular infection. LPS binding TLR4 results in the activation of transcription factor nuclear factor-kappa B (NF-κ B) [18]. NF-κB plays a major role in controlling the expression of intercellular adhesion molecule 1 (ICAM-1), interleukin-8 (IL-8) and other pro-inflammatory factors in human umbilical vein endothelial cells (HUVECs) [19]. Sheikh Rayees et al. reported that thrombin was also generated during TLR4-induced lung injury [20]. Thrombin binds a G-protein-coupled receptor, protease-activated receptor-1(PAR-1), and stimulates the GPCR-PLC pathway, resulting in calcium release from the ER [21]. However, thrombin binds to PAR-2 to generate cAMP, which abolishes calcium influx via calcium channels [20]. Thus, the signaling pathways downstream of endothelial TLR4 are critical to the pathogenesis of lung injury. In double-transgenic mice with the EC-degradation- resistant form of IκB, the cytoplasmic inhibitor of NF-κB, organ injury is decreased during LPS- or E. coli-induced peritonitis [22].

Another example is endothelial angiotensin-converting enzyme 2 (ACE2). ACE2 is now widely known as the receptor for SARS-CoV-2 (Severe Acute Respiratory Syndrome Coronavirus 2) [23], being associated with its infection. ACE2 is an essential component of the renin–angiotensin–aldosterone system (RAAS). When lung tissues are infected with SARS-CoV-2, the virus binds with the ACE2 receptor on the cell membrane and downregulates its expression, causing accumulated angiotensin II (Ang II) to activate the AT1 (losartan-sensitive receptor) [24]. Then, the PI3K-Akt signaling pathway is stimulated to regulate endothelial function and the production of IL-6 and reactive oxygen species (ROS). The decrease in ACE2 leads to a series of mechanisms that are promoted by the dysfunctional endothelium, including an increase in vascular permeability, as well as an upregulation of tissue factor. During viral infection, SARS-CoV2 binds to ACE2 to enter the cells and then endothelial cell dysfunction (ED) plays a detrimental role by worsening inflammation [25].

**Figure 1:**
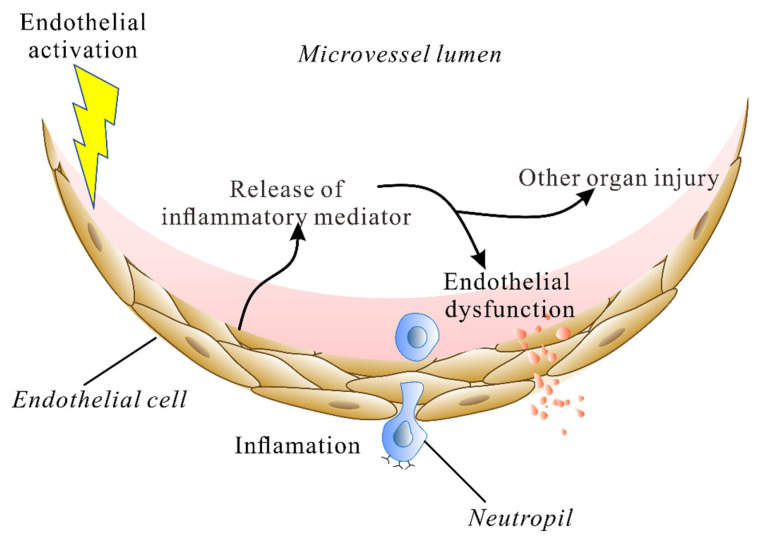
The endothelium is both a sensor of stimulation and a mediator of organ injury in ALI. The endothelium can sense intravascular stimulation and the activation of endothelium results in an increased activation of pro-inflammation transcriptions and release of inflammatory mediators, which leads to dysfunction of endothelium and other organ injury.

Therefore, the activation of the endothelium results in an increased activation of pro-inflammation transcriptions and the release of inflammatory mediators. This in turn leads to dysfunction of the endothelium and other organ injury (Figure 1). A compromised endothelial barrier during ALI increases alveolar-capillary permeability to fluid, proteins, neutrophils and red blood cells.

## 3. Calcium Influx into ECs Mediates Lung Injury

The regulation of EC permeability is mediated by various inflammatory mediators, which has been widely demonstrated to induce intracellular Ca^2+^ mobilization. It has been recognized that the increase in EC permeability is induced by rising intracellular Ca^2+^. The rise in Ca^2+^ in EC activates signal-transduction cascades that decrease the (vascular endothelial) VE-cadherin-dependent sites of cell adhesion [26]. VE-cadherin is one of the adhesion molecules that is vital for the formation and maintenance of endothelial junctions [27]. The disruption of VE-cadherin function results in interstitial edema and the accumulation of inflammatory cells in the lung microcirculation. This rise in endothelial cells [Ca^2+^]_i_ also results in the leukocyte adhesion receptor P-selectin expression [28]. EC expression of P-selectin increases leukocyte rolling on the vessel surface, which is pathogenic in lungs. VEGF, known as a major player in angiogenic pathway, has been reported to be directly involved in the regulation of EC permeability in a Ca^2+^-dependent manner [29].

Intracellular calcium signals precisely coordinate EC permeability and the inflammatory response. ECs produce a soluble form of sTNFR1 and sTNFR2 via shedding the membrane-bound molecule [30]. The functional role of sTNFR1 may include reducing the pro-inflammatory functions of TNF, which is able to activate ECs as inflammatory factors. Atrosimab, an antagonist of TNFR1, significantly reduced the TNF-mediated upregulation of the adhesion proteins ICAM-1 and VCAM-1 in a human-derived endothelial cell line [31]. Intracellular Ca^2+^ modulates TNFR1 release into the extracellular space via exosome vesicles. This shows that the precise regulation of [Ca^2+^]_i_ participates in the activation of ECs. Furthermore, some studies in humans or animal models indicated that elevated circulating calcium levels also affected EC functions [32,33].

## 4. Calcium Machinery

**Figure 2:**
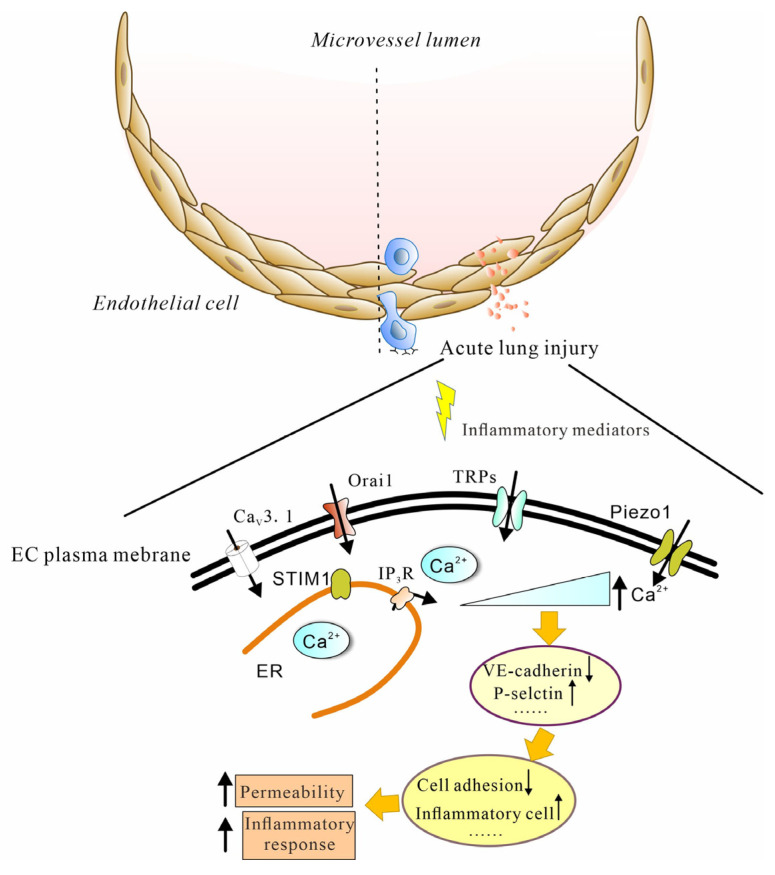
The main calcium channels involved in endothelial cell activation. The activation of ECs is accompanied by the open calcium channel. Ca^2+^ influx via these channels leads to increased permeability and other inflammatory responses.

Plasma-membrane ion channels and intracellular receptors are the main components of the complex calcium machinery. Calcium-permeant channels are potentially involved in the regulation of barrier integrity in the lung microvascular endothelium. The evidence for calcium channels being involved in regulating endothelial-barrier function during lung injury is reviewed here. During lung injury, specific acute inflammation causing endothelial dysfunction may have different harmful functional consequences, therefore it is necessary to identify the specific molecular targets for the calcium-entry-dependent regulation of endothelial function. The calcium-permeant channels involved in lung EC functions include T-type voltage-gated (VG) channels, store-operated Ca^2+^ channels (SOCs), transient-receptor-potential (TRP) channels and Piezo1 channels (Figure 2).

### 4.1. Voltage-Gated Ca^2+^ Channels

Voltage-gated calcium channels are considered key transducers of membrane potential changes for intracellular Ca^2+^ transients that modulate many physiological events [34]. ECs are usually considered non-excitable cells without VG channels. In endothelial cells, Ca^2+^ entry is typically conducted via store-operated and receptor-operated pathways. Until 2003, the T-type Ca_V_ channel Ca_V_3.1 was detected in pulmonary-macrovascular, pulmonary-artery, and endothelial cells [35,36]. T-type calcium channels are low-voltage-gated channels, which is expressed in ECs [37]. There are few reports of N-type or L-type channels. The Ca_V_3.1channel is expressed in lung microvascular endothelial cells (PMVECs), but not in lung macrovascular (e.g., pulmonary artery) cells. The channel may be activated by membrane depolarization induced by Gq-linked agonists or flow cessation [35,38]. Voltage-dependent activation indicates that the maximum current activation of the Ca_V_3.1 T-type Ca^2+^ current in PMVECs is observed at −10 mV; voltage-dependent conductance and inactivation properties suggest a “window current” in the range of −60 to −30 mV. Cav3.1 channels contribute to thrombin-induced Ca^2+^ influx in PMEVCs, whereas acetylcholine (ACh)-induced endothelium-dependent relaxation was significantly reduced in pulmonary arteries from Ca_V_3.1^−/−^ compared to wild-type mice, as well as in the presence of T-VGCC inhibitors (NNC 55-0396 or mibefradil) [35,39]. T-type Ca^2+^ channels also promote proliferation, cell-matrix adhesion, and migration of PMVECs via the α1G-CaMK4 signaling complex and the PI3K-Akt signaling pathway [37,40]. Immunofluorescence labeling revealed the presence of Ca_V_3.1 channels in endothelial cells that co-localized with endothelial nitric-oxide synthase in arteries from wild-type mice. Additionally, the downregulation of Ca_V_ also increased Ach-induced endothelial [Ca^2+^]_i_ and NO production. This study also suggests that the T-type Ca_V_ channel mediating Ca^2+^ influx may play an important role in the release of VWF (von Willebrand factor) and membrane-surface expression of P-selectin from lung microvascular endothelium, which plays an active part in inflammation [40,41].

Furthermore, the channel inhibitors mibefradil and flunarizine (classical) were found to protect mice against LPS-induced lung injury [42]. The treatment of mibefradil significantly decreased the total number of LPS-induced cells, protein concentration, and Evans-blue extravasation, as well as tumor necrosis factor alpha (TNF-α) and IL-6 levels in bronchoalveolar lavage fluid (BALF), and suppressed NF-κB activation. These data suggest that the inhibition of Ca_V_3.1 might contribute to the prevention of lung injury. However, T-type Ca_V_ channels in pulmonary microvascular endothelial cells are quite limited and the channel expression was not only limited in lung ECs. Therefore, direct evidence of EC Ca_V_3.1 in ALI needs more studies of experimental lung-injury models based on transgenic animal technology.

### 4.2. Store-Operated Calcium (SOC) Channel

SOCs are a ubiquitous Ca^2+^-entry pathway, stimulated by numerous cell surface receptors via the depletion of Ca^2+^ concentration in the ER. SOC channels are considered the major Ca^2+^-entry pathways in non-excitable cells. The STIM1 (stromal-interaction molecule) on endoplasmic reticulum (ER) and Orai1 on plasma membrane are the molecular identities responsible for SOC-channel activation. When ER Ca^2+^ stores are depleted, STIM1 proteins are promoted to aggregate and interact with Orai1 to open the channel, mediating Ca^2+^ entry [43,44]. Therefore, STIM proteins are ER Ca^2+^ sensors and Orai proteins are structural components of the SOC channel.

In EC-specific *Stim1*-knockout mice, LPS-induced vascular permeability changes were significantly reduced [45,46]. Moreover, ECs lacking Stim1 failed to trigger store-operated Ca^2+^ entry and nuclear accumulation of NFAT, a family transcription factor that influences gene expression. Similar results were obtained in human cell lines. Using a human septic serum or lipopolysaccharide (LPS)-induced human umbilical vein endothelial cell (HUVEC) experimental system, downregulation of STIM1 by RNAi or channel inhibitors significantly inhibited serum or LPS-induced extracellular calcium influx, and then the release of inflammatory cytokines [47,48]. However, one study indicated that the contribution of STIM1 to EC permeability may be independent of calcium influx [49]. In HUVECs, thrombin-mediated endothelial-barrier disruption requires STIM1 protein, but independently of Oria1 and Ca^2+^. Their results demonstrated that STIM1 protein played a key role in RhoA activation and the formation of actin stress fibers [49]. 

Pharmacological channel inhibitors also showed potential therapeutic effects in an LPS-induced ALI mouse model. BTP2, a small-molecule SOC-channel blocker, was delivered two hours after systemic LPS administration and resulted in a striking reduction in vascular leakage and pulmonary edema [45,46]. The total flavonoids from the endemic herbal medicine Nervilia fordii (Hance) exhibits potent anti-inflammatory effects and protective effects against the endotoxin lipopolysaccharide (LPS)-induced acute lung injury, and shows clinical benefits in severe acute respiratory syndromes (SARS) [50]. Rhamnocitrin, a flavonoid extracted from Nervilia fordii, repressed calcium store-operated Ca^2+^ entry induced by LPS, and exhibited the potential inhibition of endothelial activation induced by LPS [51].

### 4.3. TRP Channels

The TRP channel is a non-selective cation channel that is predominately permeable for Ca^2+^ located mostly on the plasma membranes of animal tissues [52,53]. These channels can be activated by a range of stimuli, including endogenous and exogenous chemical mediators, physical stimuli, such as temperature and mechanical forces, free cytosolic Ca^2+^ ions, the depletion of Ca^2+^ stores in the ER, and many others. Additionally, some TRP channels are voltage-sensitive and ligand-gated. TRP channels are ubiquitously present in different tissues of mammalian species. The channels mediate various physical activities, such as the sensation of pain, coldness, warmth or hotness, pressure, taste, and vision. The activation of TRP channels is associated with G-protein-coupled receptors (GPCRs), receptor tyrosine kinases, and phospholipase C. The role of TRP channels and their diverse involvement in critical pathophysiological events has been reviewed elsewhere [54,55,56].

According to their amino-acid sequence, TRP channels are categorized into six sub-families, including TRP canonical or classical (TRPC), TRP vanilloid (TRPV), TRP melastatin (TRPM), TRP ankyrin (TRPA), TRP polycystin (TRPP), and TRP mucolipin (TRPML) [53]. They are key players in the regulation of intracellular calcium. All TRP channels share a common architecture of six transmembrane-spanning helices with intracellular N- and C-termini. TRPC1, TRPC4, TRPC6, TRPM2, TRPM8, TRPV1 and TRPV4 are the most prominently expressed TRP channels in lung ECs [57], where they mediate Ca^2+^ influx and signaling. Neither the expression nor functional role(s) of any TRPML, TRPP, and TRPA family members has been studied in the lung endothelium. Considering the activation characteristics of the channel, TRP channels may act as chemosensors during toxicant induced ALI [56]. Besides increased intracellular calcium levels, the role of TRP channels in EC dysfunction during ALI is also mediated via the covalent modification of cysteine residues on the ion channel or through lipid messengers such as diacylglycerol and phosphatidylinositol phosphate [56]. It is worth noting that TRP-channel subunits can form heterotetramers. In the lung endothelium, heteromeric TRPC1/4, TRPC3/6 and TRPV1/4 are found to contribute to modulating EC permeability.

The TRPC family has been shown to play an important role in regulating Ca^2+^ entry in ECs. TLR4 or EGFR are activated by their ligands. Then, EC TRPC channels are opened by GPCR-operated activation resulting in increased intracellular Ca^2+^. Ca^2+^ binds to CaM and in turn activates MLCK. The downstream events include the activation of myosin light chain II, the interaction of actin–myosin and the formation of F-actin stress fibers. Thus, the endothelial inter-endothelial gap is formed and endothelial-barrier failure ensues.

Evidence indicates that TRPC1/4 activation can disrupt adherence junctions (AJs), resulting in endothelial hyperpermeability [58]. ECs Trpc1^−/−^ mice showed significant resistance to thrombin-induced hyperpermeability and exhibited 60% less mortality from endotoxin. A mechanistic study indicated that the loss of TRPC1 augments cell-surface VE-cadherin expression and thereby prevents AJ disruption [59]. In HUVECs, evidence also indicated that TRPC1-mediated calcium entry was associated with LPS-induced calcium overload and cell apoptosis [48].

TRPC6 is gated by C-type phospholipase (PLC) pathways and highly expressed in the lung. Lung endothelial cells from TRPC6-deficient mice showed attenuated ischemia-induced Ca^2+^ influx, cellular shape changes and impaired barrier function [60]. Norbert Weissmann et al. suggested that endothelial ROS production following ischemia–reperfusion leads to PLCγ activation and subsequent TRPC6 activation resulting in increased vascular permeability [60]. In another study, they found that LPS induces Ca^2+^ entry mediated by TRPC6 in ECs in a TLR4-dependent manner. Furthermore Ca^2+^ entry mediated by TRPC6, in turn, activates the non-muscle myosin light chain kinase (MYLK), which not only increases lung vascular permeability but also serves as a scaffold to promote the interaction of myeloid differentiation factor 88 (MyD88) and IL-1R-associated kinase 4, which are required for NF-κB activation and lung inflammation [61].

TRPV4 is a key channel for increasing endothelial permeability [62]. TRPV4-deficiency reduces hydrostatic lung edema formation and capillary leakage [63,64]. The channel can be activated by diverse stimuli including hypotonic cell swelling, deflection of the plasma membrane, warm temperatures and endogenous activators, including anandamide-derived and arachidonic-acid-derived epoxyeicosatrienoic acids [65,66]. The TRPV4 activators 4alpha-phorbol-12,13-didecanoate(4α-PDD) and 5,6- or 14,15-epoxyeicosatrienoic acid can increase lung endothelial permeability in a dose- and calcium-entry dependent manner in isolated rat lung [67]. Systemic activation of the channel also causes endothelial failure and circulatory collapse [67]. Vice versa, ruthenium red, a TRPV antagonist, blocked the permeability response induced by the TRPV4 agonists. The permeability response to 4alpha-phorbol-12,13-didecanoate was absent in TRPV4^−/−^ mice, whereas the response to thapsigargin remained unchanged [68]. Notably, The TRPV4 agonist by GSK1016790A was reported to regulate the membrane expression of the TRPV4 channel [69], which accelerates EC calcium influx, resulting in EC dysfunction. The role of TRPV4 in lung EC permeability was further confirmed in varied lung-injury models, including fluid- [70], ROS- [71], chemically [72], and even ventilator-induced lung injury [73].

Therefore, TRPV4 antagonists are the most studied compounds to reduce EC permeability (review [74]). In animal lung-injury models, some studies indicated that TRPV4 antagonists were effective in inhibiting lung EC permeability by inflammatory stimulation [8,9,75]. GSK2193874 and HC-067047, as selected TRPV4 inhibitors, showed a prophylactic effect for pulmonary edema or lung injury [64,76,77]. In lungs, GSK2193874 prevented the increased vascular permeability and resultant pulmonary edema induced by elevated pulmonary venous pressure. Two other blockers (GSK2220961 and GSK2337429) were found to show potential protection from acute lung injury when they were added 30 min after acid aspiration by gastroesophageal reflux [45]. HC-067047 was administered 45 minutes after acid instillation and showed effective prevention against TRPV4-dependent ventilator-induced lung injury in mice. Besides lung ECs, alveolar epithelial and neutrophil cells, which contribute to the pathogenesis of ALI, also have the expression of TRPV4 channels. Therefore, the protection effect of TRPV4 antagonists may have a combination effect on EC permeability, alveolar epithelial barrier function [78], and neutrophil activation [64] in lung-injury animal models. Most interestingly, TRPV4 was also proposed as a promising therapeutic target, and blocking TRPV4 may be a feasible approach to the the recent coronavirus disease 2019 (COVID-19) pandemic by protecting the alveolo-capillary barrier of the lungs from severe acute respiratory syndrome coronavirus type 2 (SARS-CoV-2) [79]. However, clinically successful drugs still need to be established. 

GlaxoSmithKline (GSK) previously reported a series of TRPV4 antagonists quinolines and benzimidazoles, but failed to deliver clinical drug candidates [80,81]. GSK2798745, an orally active TRPV4-channel blocker, is used in research for the treatment of pulmonary edema associated with congestive heart failure. This compound has been evaluated in a first-time-in-humans study (Clinicaltrials.gov identifier: NCT02119260, accessed on 26 April 2022) [82]. However, GSK2798745 failed in the phase I clinical trial to investigate the effects of the compound on alveolar barrier disruption in an LPS challenge model (NCT03511105) [83]. The results indicated that GSK2798745 did not affect segmental LPS-induced elevation of BAL total protein or neutrophils, despite blood and lung exposures. It is possible that the limited damage caused by the segmental LPS model was insufficient. In several pre-clinical models, including those mentioned above, TRPV4 blockers have been shown to increase survival, and one model resulted in the death of all intreated mice. TRPV4 might play a key role in a severe LPS-induced lung-injury model, but is less important in a light model that can recover spontaneously.

The TRPM2 channel is an oxidant-sensitive Ca^2+^-permeable channel. Oxidants generated by activated endothelial cells are known to increase vascular endothelial permeability and play a crucial role in several lung diseases. In cultured pulmonary-artery endothelial cells, TRPM2 proved to be involved in mediating oxidant-induced calcium entry and endothelial hyperpermeability [84]. In human pulmonary-artery endothelial cells (HPAECs), ROS production or directly applying ROS induced PKCα activation and phosphorylation of TRPM2 at Ser 39. This in turn stimulated a large entry of Ca^2+^ and activated the apoptosis pathway [85]. Particulate matter (PM) is pro-inflammatory to the endothelium and increases vascular permeability in vitro and in vivo via ROS generation. In a study using PM to challenge human lung ECs, it was found that PM induces marked increases in vascular permeability via ROS-mediated calcium leakage via activated TRPM2 [86]. TRPM2 deletion markedly reduced endothelial-cell apoptosis in the lungs and significantly improved the survival rate of LPS-challenged mice [85].

In summary, pro-inflammatory or barrier-disturbing receptors including TLR4 and EGFR are activated by their ligands, which open the EC GPCR-operated activation of TRPC channels, increasing intracellular Ca^2+^. TRPC1 is activated by IP3-dependet Ca^2+^ release and TRPC6 is activated by DAG. Ca^2+^ influx activates the CaM/MLCK (myosin light chain kinase)-signaling pathway by binding to CaM, which in turn activates MLCK. Activated MLCK inhibits MLCP, thereby activating MLC II (myosin light chain II), the actin–myosin interaction and the formation of F-actin stress fibers. The resulting tensile force on endothelial cell–cell junctions (inside-out signaling) causes inter-endothelial gap formation and endothelial-barrier failure.

Besides increased intracellular calcium levels, the role of TRP channels in EC dysfunction during ALI are also mediated through the covalent modification of cysteine residues on the ion channel or through lipid messengers, such as diacylglycerol and phosphatidylinositol phosphate.

### 4.4. Piezo Channel

The Piezo channel is a mechanically activated ion channel involved in sensing forces in various cell types and tissues. In 2010, the Piezo family was discovered and named. During the past 10 years, Piezo1 was found mainly to be expressed in non-excitable cell types in experimental animal models, while Piezo2 is expressed in sensory neurons [87]. Piezo channels are mainly permeable to Ca^2+^ but also to other cations, such as Na^+^, K^+^, and Mg^2+^, therefore being non-selective Ca^2+^ channels. Studies showed that piezo1 plays an important role in various physical activities, including vascular function, epithelial homeostasis, red-blood-cell-volume regulation, etc. Piezo1 is activated by several mechanical stress forces such as physiological shear stress and cell-wall tension.

Calcium influx is mediated by Piezo transduction of mechanical signals into biological signals [88]. Recently, the contribution of the Piezo1 channel was reported to be involved in regulating EC function. It has been demonstrated that Piezo1 is the mechanical sensor responsible for hydrostatic pressure-induced endothelial-barrier breakdown [89], ventilator-induced lung injury [90,91,92] and radiation-induced lung injury [93].

Lung-endothelial-barrier dysfunction and edema induced by raising pulmonary microvessel pressure were abolished in EC_Piezo1_-KOmice, in which Piezo1 was conditionally deleted in ECs [89]. The deficiency or inhibition of Piezo1 by GSMTx4 dramatically alleviated VILI-induced pathologic changes and improved the seven-day mortality rate in the model rats [92]. In an ionizing-radiation (IR)-induced injury model, Piezo1 protein expression was upregulated in vivo and in vitro [93]. The upregulation of Piezo1 by injury stimulations or the channel agonist Yoda1 leads to an increased influx of Ca^2+^, which increases calpain activity and the degradation of VE-cadherin. Therefore, Piezo1 probably mediates lung vascular hyperpermeability by promoting the internalization and degradation of the endothelial adherence-junction (AJ) protein VE-cadherin.

Piezo1 and TRPV4 channels have both, independently, been involved in high-venous-pressure- and fluid-shear-stress-induced lung microvascular hyperpermeability in endothelial cells. Sandip M Swain and Rodger A Liddle investigated the mechanism that Piezo1 and TRPV4 channels perform during endothelial dysfunction [73]. HC067047, the TRPV4 channel antagonist, was able to prevent the loss of endothelial-cell integrity induced by the activation of Piezo1. The results indicated that Piezo1 activation by fluid shear stress initiates a calcium signal that causes TRPV4 opening, which in turn is responsible for the sustained phase of calcium elevation that triggers pathological events in endothelial cells.

### 4.5. Calcium Flux: A New Target for Therapy

Calcium influx plays a crucial role in the occurrence and development of ALI. An increase in intracellular calcium not only increases the permeability of endothelial cells, but also contributes to neutrophil activation and endothelial cellular inflammation. Therefore, blocking calcium influx into ECs is a potential therapeutic strategy for lung injury. Some calcium-channel modulators were effective in inhibiting lung EC permeability, as well as in lung-injury mouse models. The compounds mentioned in this article are shown in Table 1.

## 5. Conclusions

The key role of calcium influx in ALI has been illuminated by the accumulated studies. Increased intracellular calcium promotes the transformation of EC morphology and the expansion of AJs, which leads to the increased permeability of ECs. The calcium-signaling cascade also contributes to the endothelial cellular inflammatory response, such as the increased expression of vascular adhesion molecules. Therefore, targeting calcium influx into cells is an effective strategy for ALI therapy and some calcium-channel blockers significantly reduce the increase in endothelial permeability and inflammation. Evidence obtained from these studies provides a convincing rationale for human therapies that target the EC dysfunction during ALI, especially in the early phase of ALI. However, several important points still need to be addressed before calcium-channel blockers can be used to treat human lung injury. First, these calcium channels such as TRPV4 channels are not only expressed in EC but also other cell types. Channel blockers may have off-target effects, especially on immune cells. Second, most lung-injury models used in evaluating the protection effect of channel blockers are induced by LPS. The channel-antagonist-mediated inhibition of lung EC dysfunction should be tested in the presence of a live bacterial infection, which is more comparable to innate or adaptive immune response to infection. Finally, although modulators for calcium channels are the pots of gold for anti-ALI development, high quality and widely available channel modulators are not many, and modulators should be further generated with properties that make them suitable for in vivo use.

## Figures and Tables

**Table 1:** Calcium-channel modulators with potential blockage of EC permeability during ALI.

Target Channel	Compounds	Application	Reference
Ca_V_3.1	Mibefradil	LPS-induced lung injury	[36,42]
	Flunarizine	LPS-induced lung injury	[36,42]
SOCs	Rhamnocitrin	A potent inhibitor of endothelial activation	[51]
TRPV4	GSK2798745	LPS-induced lung inflammation *	[77,84]
	GSK2193874	High-pulmonary-venous-pressure (PVP)-induced edema	[77]
	HC-067047	LPS-induced mouse lung injury	[9]
Piezo1	GSMTx4	Ionizing-radiation (IR)-induced lung injury	[93]

* Failed in Phase I, Clinicaltrials.gov identifier: NCT03511105, accessed on 26 April 2022. Others are performed in animal model.
